# Supplementary material for: APOE4 is associated with elevated blood lipids and lower levels of innate immune biomarkers in a tropical Amerindian subsistence population
Source: eLife. 2021 Sep 29;10:e68231. doi: 10.7554/eLife.68231 (PMC8480980; doi:10.7554/eLife.68231)
Supplement: Supplementary file 1. [file elife-68231-supp1.docx]

|  | **CRP (mg/L)** | | **Eosin : Lymph** | | **Sed Rate (mm/hr)** | | **Lymphocytes (mm^3^)** | | **Neutrophils (mm^3^)** | | **Eosinophils (mm^3^)** | |
| --- | --- | --- | --- | --- | --- | --- | --- | --- | --- | --- | --- | --- |
| *Predictors* | *Estimate (95% CI)* | *p* | *Estimate (95% CI)* | *p* | *Estimate (95% CI)* | *p* | *Estimate (95% CI)* | *p* | *Estimate (95% CI)* | *p* | *Estimate (95% CI)* | *p* |
| (Intercept) | -2.74 | **<0.001** | 0.72 | **<0.001** | 5.9 | **<0.001** | 35.1 | **<0.001** | 63.37 | **<0.001** | 58.59 | **<0.001** |
|  | (-4.09 – -1.38) |  | (0.61 – 0.83) |  | (4.59 – 7.21) |  | (29.32 – 40.88) |  | (55.84 – 70.89) |  | (49.82 – 67.37) |  |
| APOE [E4] | -0.55 | 0.147 | -0.06 | **0.021** | -0.04 | 0.911 | 1.17 | 0.424 | -2.89 | 0.125 | -5.07 | **0.022** |
|  | (-1.29 – 0.19) |  | (-0.12 – -0.01) |  | (-0.69 – 0.62) |  | (-1.70 – 4.04) |  | (-6.58 – 0.80) |  | (-9.40 – -0.74) |  |
| Age [in years] | 0.01 | **<0.001** | 0 | **<0.001** | 0.01 | **<0.001** | -0.07 | **<0.001** | 0.05 | **0.003** | -0.2 | **<0.001** |
|  | (0.01 – 0.02) |  | (-0.00 – -0.00) |  | (0.01 – 0.02) |  | (-0.10 – -0.05) |  | (0.02 – 0.08) |  | (-0.24 – -0.17) |  |
| Sex [women] | 0.01 | 0.794 | 0 | 0.777 | 1.07 | **<0.001** | 1.03 | **<0.001** | -0.52 | 0.115 | 0.89 | **0.026** |
|  | (-0.09 – 0.12) |  | (-0.01 – 0.01) |  | (0.95 – 1.19) |  | (0.51 – 1.54) |  | (-1.17 – 0.13) |  | (0.11 – 1.67) |  |
| BMI | 0.89 | **<0.001** | -0.08 | **<0.001** | -0.69 | **0.001** | 5.82 | **<0.001** | 1.08 | 0.345 | -3.09 | **0.02** |
|  | (0.49 – 1.29) |  | (-0.12 – -0.05) |  | (-1.08 – -0.30) |  | (4.09 – 7.54) |  | (-1.16 – 3.33) |  | (-5.70 – -0.48) |  |
| Season [wet] | 0.16 | **0.018** | -0.01 | **0.002** | 0.32 | **<0.001** | -1.19 | **<0.001** | -1.02 | **<0.001** | -1.83 | **<0.001** |
|  | (0.03 – 0.29) |  | (-0.02 – -0.00) |  | (0.24 – 0.40) |  | (-1.57 – -0.81) |  | (-1.55 – -0.50) |  | (-2.40 – -1.26) |  |
| high WBC | 0.46 | **<0.001** | 0.05 | **<0.001** | 0.4 | **<0.001** | 8.41 | **<0.001** | 21.85 | **<0.001** | 12.25 | **<0.001** |
|  | (0.22 – 0.70) |  | (0.04 – 0.07) |  | (0.25 – 0.55) |  | (7.69 – 9.13) |  | (20.86 – 22.83) |  | (11.17 – 13.32) |  |
| E4 * Age | 0 | 0.481 | 0 | 0.096 | 0 | 0.634 | -0.02 | 0.375 | 0.05 | 0.169 | 0.06 | 0.14 |
|  | (-0.01 – 0.02) |  | (-0.00 – 0.00) |  | (-0.01 – 0.01) |  | (-0.08 – 0.03) |  | (-0.02 – 0.11) |  | (-0.02 – 0.14) |  |
| **Random Effects** | | | | | | | | | | | | |
| Residual σ^2^ | 0.78 | | 0.01 | | 1.97 | | 46.72 | | 90.48 | | 103.3 | |
| τ_00_ | 0.01 _pid_ | | 0.00 _pid_ | | 0.68 _pid_ | | 10.23 _pid_ | | 13.11 _pid_ | | 23.89 _pid_ | |
|  | 0.03 _community_id_ | | 0.00 _community_id_ | | 0.11 _community_id_ | | 1.36 _community_id_ | | 3.68 _community_id_ | | 9.35 _community_id_ | |
| ICC | 0.04 | | 0.25 | | 0.28 | | 0.2 | | 0.16 | | 0.24 | |
| N | 908 _pid_ | | 1260 _pid_ | | 1254 _pid_ | | 1260 _pid_ | | 1260 _pid_ | | 1260 _pid_ | |
|  | 68 _community_id_ | | 80 _community_id_ | | 80 _community_id_ | | 80 _community_id_ | | 80 _community_id_ | | 80 _community_id_ | |
| Observations | 1033 | | 6121 | | 5988 | | 6136 | | 6145 | | 6130 | |
| Marginal R^2^ / Conditional R^2^ | 0.072 / 0.113 | | 0.036 / 0.273 | | 0.113 / 0.366 | | 0.107 / 0.285 | | 0.235 / 0.355 | | 0.114 / 0.329 | |

**Supplementary File 1a.** Models showing immune function and age associations, including interactive effects with *APOE* status. Results are from mixed effects linear regressions, adjusting for sex, season, and a dummy variable used as a proxy for current illness (leukocytes > 12 mm3). Results are reported as standardized betas; *CI* is the 95% confidence interval. All dependent variables were transformed and centered prior to analyses. See methods section for transformations. *APOE* genotype is coded as a categorical variable, binned as individuals that are homozygous *E3* (E3) versus those that have at least one copy of the *E4* allele (E4).

|  | **CRP (mg/L)** | | | **Sed Rate (mm/hr)** | | **Neutrophils (mm^3^)** | | **Eosinophils (mm^3^)** | | **Eosinophil : Lymphocyte ratio** | |
| --- | --- | --- | --- | --- | --- | --- | --- | --- | --- | --- | --- |
| *Predictors* | *β ( 95%CI )* | | *p* | *β ( 95%CI )* | *p* | *β ( 95%CI )* | *p* | *β ( 95%CI )* | *p* | *β ( 95%CI )* | *p* |
| (Intercept) | -0.80 (-1.14 – -0.46) | | **<0.001** | -0.87 (-1.04 – -0.70) | **<0.001** | -0.27 (-0.41 – -0.13) | **<0.001** | 0.82 (0.66 – 0.99) | **<0.001** | 0.60 (0.42 – 0.77) | **<0.001** |
| *APOE4* | -0.29 (-0.44 – -0.14) | | **<0.001** | 0.06 (-0.03 – 0.15) | 0.182 | -0.03 (-0.10 – 0.04) | 0.435 | -0.16 (-0.24 – -0.08) | **<0.001** | -0.14 (-0.23 – -0.06) | **0.001** |
| Sex [women] | 0.01 (-0.11 – 0.13) | | 0.863 | 0.60 (0.53 – 0.67) | **<0.001** | -0.05 (-0.10 – 0.01) | 0.095 | 0.07 (0.01 – 0.13) | **0.032** | 0.01 (-0.06 – 0.07) | 0.859 |
| Age [years] | 0.01 (0.01 – 0.02) | | **<0.001** | 0.01 (0.01 – 0.01) | **<0.001** | 0.00 (0.00 – 0.01) | **<0.001** | -0.01 (-0.02 – -0.01) | **<0.001** | -0.01 (-0.01 – -0.01) | **<0.001** |
| high WBC | 0.43 (0.17 – 0.68) | | **0.001** | 0.23 (0.15 – 0.32) | **<0.001** | 1.82 (1.73 – 1.90) | **<0.001** | 0.98 (0.90 – 1.07) | **<0.001** | 0.37 (0.28 – 0.46) | **<0.001** |
| season [wet] | 0.15 (0.01 – 0.29) | | **0.040** | 0.18 (0.14 – 0.23) | **<0.001** | -0.09 (-0.13 – -0.04) | **<0.001** | -0.14 (-0.19 – -0.10) | **<0.001** | -0.07 (-0.12 – -0.02) | **0.004** |
| **Random Effects** | | | | | | | | | | | |
| Residual σ^2^ | 0.90 | | | 0.64 | | 0.63 | | 0.66 | | 0.73 | |
| τ_00_ | 0.04 _pid_ | | | 0.22 _pid_ | | 0.09 _pid_ | | 0.15 _pid_ | | 0.20 _pid_ | |
|  | 0.03 _community_id_ | | | 0.03 _community_id_ | | 0.03 _community_id_ | | 0.06 _community_id_ | | 0.05 _community_id_ | |
| ICC | 0.07 | | | 0.28 | | 0.16 | | 0.25 | | 0.26 | |
| N | 909 _pid_ | | | 1256 _pid_ | | 1262 _pid_ | | 1262 _pid_ | | 1262 _pid_ | |
|  | 68 _community_id_ | | | 80 _community_id_ | | 80 _community_id_ | | 80 _community_id_ | | 80 _community_id_ | |
| Observations | 1034 | | | 5992 | | 6149 | | 6134 | | 6125 | |
| Marginal R^2^ / Conditional R^2^ | | 0.054 / 0.125 | | 0.111 / 0.364 | | 0.235 / 0.354 | | 0.111 / 0.329 | | 0.028 / 0.276 | |

**Supplementary File 1b.** Mixed effects linear regressions assessing the association between *APOE* genotype and measures of innate immune function. The main predictor of interest, *APOE4*, is a binary variable that represents having at least a single copy of the *E4* allele. All dependent variables were transformed and centered for analyses. See methods section for transformations. Random effects are included for individual and community residence. Results are reported as standardized betas (2.5% - 97.5% confidence intervals).

|  | **Leukocytes (1000/mm^3^)** | | | **Lymphocytes (mm^3^)** | | | **Hemoglobin (mg/dL)** | | |  |  |
| --- | --- | --- | --- | --- | --- | --- | --- | --- | --- | --- | --- |
| *Predictors* | *β ( 95%CI )* | | *p* | *β ( 95%CI )* | | *p* | *β ( 95%CI )* | | *p* |  |  |
| (Intercept) | 0.23 (0.10 – 0.37) | | **0.001** | 0.51 (0.35 – 0.66) | | **<0.001** | 1.40 (1.23 – 1.57) | | **<0.001** |  |  |
| *APOE4* | -0.08 (-0.14 – -0.01) | | **0.025** | 0.01 (-0.08 – 0.09) | | 0.892 | -0.04 (-0.13 – 0.05) | | 0.400 |  |  |
| Sex [women] | 0.04 (-0.01 – 0.10) | | 0.114 | 0.13 (0.06 – 0.19) | | **<0.001** | -0.71 (-0.79 – -0.64) | | **<0.001** |  |  |
| Age [years] | -0.01 (-0.01 – -0.00) | | **<0.001** | -0.01 (-0.01 – -0.01) | | **<0.001** | -0.02 (-0.02 – -0.01) | | **<0.001** |  |  |
| high WBC | 2.01 (1.93 – 2.08) | | **<0.001** | 1.02 (0.93 – 1.10) | | **<0.001** | -0.12 (-0.20 – -0.04) | | **0.002** |  |  |
| season [wet] | -0.16 (-0.19 – -0.12) | | **<0.001** | -0.15 (-0.20 – -0.11) | | **<0.001** | -0.29 (-0.33 – -0.24) | | **<0.001** |  |  |
| **Random Effects** | | | | | | | | | | | |
| Residual σ^2^ | | 0.47 | | | 0.70 | | | 0.52 | | |  |
| τ_00_ | | 0.12 _pid_ | | | 0.17 _pid_ | | | 0.32 _pid_ | | |  |
|  | | 0.04 _community_id_ | | | 0.02 _community_id_ | | | 0.02 _community_id_ | | |  |
| ICC | | 0.25 | | | 0.21 | | | 0.39 | | |  |
| N | | 1266 _pid_ | | | 1262 _pid_ | | | 1265 _pid_ | | |  |
|  | | 80 _community_id_ | | | 80 _community_id_ | | | 80 _community_id_ | | |  |
| Observations | | 6229 | | | 6140 | | | 6199 | | |  |
| Marginal R^2^ / Conditional R^2^ | | 0.315 / 0.483 | | | 0.099 / 0.289 | | | 0.175 / 0.497 | | |  |

**Supplementary File 1c.** Mixed effects linear regressions assessing the association between *APOE* genotype and other measures of immune function. The main predictor of interest, *APOE4*, is a binary variable that represents having at least a single copy of the *E4* allele. All dependent variables were transformed and centered for analyses. See methods section for transformations. Random effects are included for individual and community residence. Results are reported as standardized betas (2.5% - 97.5% confidence intervals).

|  | **CRP (< 10mg/L)** | | **CRP (< 5mg/L)** | | **CRP (< 3mg/L)** | |
| --- | --- | --- | --- | --- | --- | --- |
| *Predictors* | *β ( 95%CI )* | *p* | *β ( 95%CI )* | *p* | *β ( 95%CI )* | *p* |
| (Intercept) | -0.75 (-1.08 – -0.42) | **<0.001** | -0.69 (-1.01 – -0.36) | **<0.001** | -0.94 (-1.27 – -0.61) | **<0.001** |
| *APOE4* | -0.23 (-0.38 – -0.09) | **0.001** | -0.22 (-0.36 – -0.08) | **0.002** | -0.14 (-0.28 – -0.01) | **0.036** |
| Sex [women] | -0.03 (-0.15 – 0.08) | 0.603 | -0.01 (-0.12 – 0.11) | 0.918 | 0.02 (-0.09 – 0.13) | 0.739 |
| Age [years] | 0.01 (0.01 – 0.02) | **<0.001** | 0.01 (0.00 – 0.01) | **0.013** | 0.00 (-0.00 – 0.01) | 0.178 |
| high WBC | 0.36 (0.11 – 0.62) | **0.005** | 0.31 (0.10 – 0.52) | **0.004** | 0.21 (-0.03 – 0.44) | 0.087 |
| season [wet] | 0.08 (-0.06 – 0.21) | 0.271 | -0.07 (-0.20 – 0.06) | 0.296 | -0.00 (-0.13 – 0.13) | 0.963 |
| **Random Effects** | | | | | | |
| σ^2^ | 0.81 | | 0.57 | | 0.33 | |
| τ_00_ | 0.02 _pid_ | | 0.06 _pid_ | | 0.11 _pid_ | |
|  | 0.03 _community_id_ | | 0.02 _community_id_ | | 0.02 _community_id_ | |
| ICC | 0.05 | | 0.12 | | 0.27 | |
| N | 860 _pid_ | | 706 _pid_ | | 510 _pid_ | |
|  | 67 _community_id_ | | 65 _community_id_ | | 62 _community_id_ | |
| Observations | 979 | | 789 | | 552 | |
| Marginal R^2^ / Conditional R^2^ | 0.041 / 0.089 | | 0.034 / 0.153 | | 0.017 / 0.285 | |

**Supplementary File 1d.** Mixed effects linear regressions assessing the association between *APOE* genotype and C-reactive protein (CRP) with different cutoffs. The main predictor of interest, *APOE4*, is a binary variable that represents having at least a single copy of the *E4* allele. All dependent variables were transformed and centered for analyses. See methods section for transformations. Random effects are included for individual and community residence. Results are reported as standardized betas (2.5% - 97.5% confidence intervals).

|  | **BMI (kg/m^2^)** | | **Total Cholesterol (mg/dL)** | | **LDL (mg/L)** | | **HDL (mg/dL)** | | **oxLDL (IU/L)** | | **Troglycerides (mg/dL)** | |
| --- | --- | --- | --- | --- | --- | --- | --- | --- | --- | --- | --- | --- |
| *Predictors* | *β ( 95%CI )* | *p* | *β ( 95%CI )* | *p* | *β ( 95%CI )* | *p* | *β ( 95%CI )* | *p* | *β ( 95%CI )* | *p* | *β ( 95%CI )* | *p* |
| (Intercept) | 0.33 (0.16 – 0.50) | **<0.001** | -0.59 (-0.83 – -0.34) | **<0.001** | -0.58 (-0.83 – -0.34) | **<0.001** | -0.50 (-0.74 – -0.26) | **<0.001** | 0.31 (-0.06 – 0.68) | 0.100 | -0.15 (-0.40 – 0.09) | 0.223 |
| *APOE4* | 0.15 (0.02 – 0.28) | **0.019** | 0.15 (0.04 – 0.27) | **0.009** | 0.08 (-0.04 – 0.19) | 0.185 | 0.05 (-0.06 – 0.16) | 0.396 | 0.16 (-0.00 – 0.32) | 0.051 | 0.07 (-0.05 – 0.19) | 0.255 |
| Sex [women] | 0.05 (-0.05 – 0.16) | 0.287 | 0.15 (0.06 – 0.24) | **0.001** | 0.19 (0.10 – 0.28) | **<0.001** | -0.07 (-0.16 – 0.01) | 0.091 | 0.08 (-0.05 – 0.21) | 0.210 | 0.11 (0.02 – 0.21) | **0.022** |
| Age [years] | -0.01 (-0.01 – -0.00) | **<0.001** | 0.01 (0.00 – 0.01) | **<0.001** | 0.01 (0.01 – 0.01) | **<0.001** | 0.01 (0.00 – 0.01) | **<0.001** | -0.00 (-0.01 – 0.00) | 0.177 | 0.00 (-0.00 – 0.00) | 0.738 |
| high WBC | -0.08 (-0.11 – -0.06) | **<0.001** | -0.13 (-0.21 – -0.05) | **0.002** | -0.31 (-0.40 – -0.22) | **<0.001** | 0.07 (-0.01 – 0.16) | 0.104 | -0.32 (-0.48 – -0.16) | **<0.001** | 0.09 (0.02 – 0.17) | **0.014** |
| season [wet] | -0.01 (-0.06 – 0.04) | 0.624 | -0.01 (-0.16 – 0.15) | 0.938 | -0.06 (-0.22 – 0.09) | 0.436 | -0.07 (-0.23 – 0.09) | 0.391 | 0.07 (-0.18 – 0.31) | 0.596 | 0.04 (-0.10 – 0.18) | 0.574 |
| **Random Effects** | | | | | | | | | | | | |
| Residual σ^2^ | 0.18 | | 0.70 | | 0.71 | | 0.82 | | 0.30 | | 0.57 | |
| τ_00_ | 0.78 _pid_ | | 0.24 _pid_ | | 0.18 _pid_ | | 0.14 _pid_ | | 0.65 _pid_ | | 0.39 _pid_ | |
|  | 0.02 _community_id_ | | 0.09 _community_id_ | | 0.11 _community_id_ | | 0.10 _community_id_ | | 0.11 _community_id_ | | 0.05 _community_id_ | |
| ICC | 0.82 | | 0.32 | | 0.29 | | 0.22 | | 0.72 | | 0.44 | |
| N | 1264 _pid_ | | 1171 _pid_ | | 1157 _pid_ | | 1169 _pid_ | | 909 _pid_ | | 1176 _pid_ | |
|  | 80 _community_id_ | | 71 _community_id_ | | 71 _community_id_ | | 71 _community_id_ | | 68 _community_id_ | | 71 _community_id_ | |
| Observations | 6225 | | 2584 | | 2393 | | 2479 | | 1034 | | 2635 | |
| Marginal R^2^ / Conditional R^2^ | 0.011 / 0.818 | | 0.021 / 0.335 | | 0.043 / 0.323 | | 0.011 / 0.233 | | 0.030 / 0.725 | | 0.006 / 0.439 | |

**Supplementary File 1e.** Mixed effects linear regressions assessing the association between *APOE* genotype and measures of lipids and BMI. The main predictor of interest, *APOE4*, is a binary variable that represents having at least a single copy of the *E4* allele. All dependent variables were transformed and centered for analyses. See methods section for transformations. Random effects are included for individual and community residence. Results are reported as standardized betas (2.5% - 97.5% confidence intervals).

|  | **CRP (mg/L)** | | **Sed Rate (mm/hr)** | | **Neutrophils (mm^3^)** | |
| --- | --- | --- | --- | --- | --- | --- |
| *Predictors* | *Estimates (95% CI)* | *p* | *Estimates (95% CI)* | *p* | *Estimates (95% CI)* | *p* |
| (Intercept) | -0.76 | **<0.001** | -1.35 | **<0.001** | -0.3 | **0.003** |
|  | (-1.14 – -0.38) |  | (-1.58 – -1.13) |  | (-0.50 – -0.10) |  |
| Total Cholesterol | -0.13 | **0.001** | -0.06 | **0.002** | 0.02 | 0.216 |
|  | (-0.20 – -0.05) |  | (-0.10 – -0.02) |  | (-0.01 – 0.06) |  |
| BMI | 0.09 | **0.011** | -0.04 | 0.06 | 0.01 | 0.785 |
|  | (0.02 – 0.16) |  | (-0.08 – 0.00) |  | (-0.03 – 0.04) |  |
| Sex [women] | 0.07 | 0.279 | 0.59 | **<0.001** | -0.05 | 0.15 |
|  | (-0.06 – 0.20) |  | (0.50 – 0.67) |  | (-0.13 – 0.02) |  |
| Age ([in yrs] | 0.01 | **<0.001** | 0.02 | **<0.001** | 0.01 | **<0.001** |
|  | (0.01 – 0.02) |  | (0.01 – 0.02) |  | (0.00 – 0.01) |  |
| Current illness | 0.65 | **<0.001** | 0.17 | **0.019** | 1.83 | **<0.001** |
|  | (0.37 – 0.93) |  | (0.03 – 0.32) |  | (1.70 – 1.97) |  |
| Season [wet] | 0.13 | 0.098 | 0.18 | **<0.001** | -0.1 | **0.009** |
|  | (-0.02 – 0.29) |  | (0.10 – 0.26) |  | (-0.17 – -0.02) |  |
| Total Cholesterol * BMI | 0.15 | **<0.001**  **FDR p<0.001** | 0.05 | **0.008 FDR p=0.018** | 0.02 | 0.193 FDR p=0.248 |
|  | (0.08 – 0.21) |  | (0.01 – 0.08) |  | (-0.01 – 0.05) |  |
| **Random Effects** | | | | | | |
| Residual σ^2^ | 0.92 | | 0.63 | | 0.6 | |
| τ_00_ | 0.00 _pid_ | | 0.20 _pid_ | | 0.10 _pid_ | |
|  | 0.04 _community_id_ | | 0.05 _community_id_ | | 0.04 _community_id_ | |
| ICC | 0.04 | | 0.29 | | 0.19 | |
| N | 785 _pid_ | | 1153 _pid_ | | 1166 _pid_ | |
|  | 65 _community_id_ | | 71 _community_id_ | | 71 _community_id_ | |
| Observations | 900 | | 2530 | | 2562 | |
| Marginal R^2^ / Conditional R^2^ | 0.086 / 0.120 | | 0.131 / 0.384 | | 0.220 / 0.367 | |

**Supplementary File 1f.** Mixed effects linear regression models that test direct and interactive effects of total cholesterol and BMI on inflammatory immune markers. Random effects are included for individual and community residence. Dependent and predictor variables of interest (total cholesterol, BMI, and immune markers) were transformed and centered for analyses. See methods section for transformations. Results are reported as standardized betas (2.5% - 97.5% confidence intervals).

|  | **CRP (mg/L)** | | **Sed Rate (mm/hr)** | | **Neutrophils (mm^3^)** | |
| --- | --- | --- | --- | --- | --- | --- |
| *Predictors* | *Estimates (95% CI)* | *p* | *Estimates (95% CI)* | *p* | *Estimates (95% CI)* | *p* |
| (Intercept) | -0.75 | **<0.001** | -1.35 | **<0.001** | -0.31 | **0.003** |
|  | (-1.13 – -0.37) |  | (-1.59 – -1.12) |  | (-0.52 – -0.10) |  |
| LDL Cholesterol | -0.11 | **0.006** | -0.03 | 0.139 | 0.03 | 0.098 |
|  | (-0.18 – -0.03) |  | (-0.07 – 0.01) |  | (-0.01 – 0.07) |  |
| BMI | 0.06 | 0.099 | -0.03 | 0.131 | 0.01 | 0.708 |
|  | (-0.01 – 0.14) |  | (-0.08 – 0.01) |  | (-0.03 – 0.04) |  |
| Sex [women] | 0.06 | 0.363 | 0.58 | **<0.001** | -0.05 | 0.185 |
|  | (-0.07 – 0.19) |  | (0.50 – 0.67) |  | (-0.13 – 0.02) |  |
| Age ([in yrs] | 0.01 | **<0.001** | 0.02 | **<0.001** | 0.01 | **0.001** |
|  | (0.01 – 0.02) |  | (0.01 – 0.02) |  | (0.00 – 0.01) |  |
| Current illness | 0.65 | **<0.001** | 0.16 | **0.042** | 1.82 | **<0.001** |
|  | (0.37 – 0.93) |  | (0.01 – 0.30) |  | (1.68 – 1.96) |  |
| Season [wet] | 0.09 | 0.249 | 0.17 | **<0.001** | -0.05 | 0.224 |
|  | (-0.07 – 0.26) |  | (0.09 – 0.26) |  | (-0.12 – 0.03) |  |
| LDL Cholesterol * BMI | 0.16 | **<0.001 FDR p<0.001** | 0.03 | 0.073 FDR p=0.131 | 0.01 | 0.745 FDR p=0.745 |
|  | (0.10 – 0.22) |  | (-0.00 – 0.07) |  | (-0.03 – 0.04) |  |
| **Random Effects** | | | | | | |
| Residual σ^2^ | 0.92 | | 0.63 | | 0.6 | |
| τ_00_ | 0.00 _pid_ | | 0.21 _pid_ | | 0.11 _pid_ | |
|  | 0.04 _community_id_ | | 0.05 _community_id_ | | 0.04 _community_id_ | |
| ICC |  | | 0.29 | | 0.2 | |
| N | 779 _pid_ | | 1138 _pid_ | | 1152 _pid_ | |
|  | 65 _community_id_ | | 71 _community_id_ | | 71 _community_id_ | |
| Observations | 895 | | 2342 | | 2381 | |
| Marginal R^2^ / Conditional R^2^ | 0.087 / 0.137 | | 0.126 / 0.384 | | 0.218 / 0.371 | |

**Supplementary File 1g.** Mixed effects linear regression models that test direct and interactive effects of LDL cholesterol and BMI on inflammatory immune markers. Random effects are included for individual and community residence. Dependent and predictor variables of interest (LDL cholesterol, BMI, and immune markers) were transformed and centered for analyses. See methods section for transformations. Results are reported as standardized betas (2.5% - 97.5% confidence intervals).

|  | **CRP (mg/L)** | | **Sed Rate (mm/hr)** | | **Neutrophils (mm^3^)** | |
| --- | --- | --- | --- | --- | --- | --- |
| *Predictors* | *Estimates (95% CI)* | *p* | *Estimates (95% CI)* | *p* | *Estimates (95% CI)* | *p* |
| (Intercept) | -0.99 | **<0.001** | -1.12 | **<0.001** | -0.44 | **0.003** |
|  | (-1.33 – -0.65) |  | (-1.45 – -0.78) |  | (-0.72 – -0.15) |  |
| Oxidized LDL | 0 | 0.963 | 0.01 | 0.825 | -0.01 | 0.607 |
|  | (-0.06 – 0.06) |  | (-0.05 – 0.06) |  | (-0.06 – 0.04) |  |
| BMI | 0.1 | **0.002** | 0.01 | 0.66 | 0.01 | 0.667 |
|  | (0.04 – 0.16) |  | (-0.05 – 0.07) |  | (-0.04 – 0.06) |  |
| Sex [women] | 0.01 | 0.929 | 0.48 | **<0.001** | -0.09 | 0.073 |
|  | (-0.11 – 0.12) |  | (0.37 – 0.59) |  | (-0.18 – 0.01) |  |
| Age ([in yrs] | 0.02 | **<0.001** | 0.01 | **<0.001** | 0.01 | **<0.001** |
|  | (0.01 – 0.02) |  | (0.01 – 0.02) |  | (0.01 – 0.02) |  |
| Current illness | 0.52 | **<0.001** | 0.25 | **0.036** | 1.9 | **<0.001** |
|  | (0.26 – 0.78) |  | (0.02 – 0.49) |  | (1.69 – 2.11) |  |
| Season [wet] | 0.15 | **0.035** | -0.19 | **0.016** | 0.19 | **0.004** |
|  | (0.01 – 0.30) |  | (-0.34 – -0.03) |  | (0.06 – 0.32) |  |
| Oxidized LDL * BMI | 0.14 | **<0.001 FDR p<0.001** | -0.04 | 0.148 FDR p=0.222 | 0.02 | 0.366  FDR p=0.411 |
|  | (0.08 – 0.19) |  | (-0.09 – 0.01) |  | (-0.02 – 0.07) |  |
| **Random Effects** | | | | | | |
| Residual σ^2^ | 0.92 | | 0.54 | | 0.53 | |
| τ_00_ | 0.01 _pid_ | | 0.16 _pid_ | | 0.05 _pid_ | |
|  | 0.03 _community_id_ | | 0.13 _community_id_ | | 0.07 _community_id_ | |
| ICC | 0.04 | | 0.35 | | 0.19 | |
| N | 907 _pid_ | | 845 _pid_ | | 904 _pid_ | |
|  | 68 _community_id_ | | 68 _community_id_ | | 68 _community_id_ | |
| Observations | 1032 | | 967 | | 1027 | |
| Marginal R^2^ / Conditional R^2^ | 0.077 / 0.115 | | 0.094 / 0.415 | | 0.272 / 0.409 | |

**Supplementary File 1h.** Mixed effects linear regression models that test direct and interactive effects of oxidized LDL and BMI on inflammatory immune markers. Random effects are included for individual and community residence. Dependent and predictor variables of interest (oxidized LDL, BMI, and immune markers) were transformed and centered for analyses. See methods section for transformations. Results are reported as standardized betas (2.5% - 97.5% confidence intervals).

|  | **Total Cholesterol (mg/dL)** | | | **LDL (mg/dL)** | | | **Ox-LDL (IU/L)** | | | **HDL (mg/dL)** | | | **Triglycerides (mg/dL)** | | |
| --- | --- | --- | --- | --- | --- | --- | --- | --- | --- | --- | --- | --- | --- | --- | --- |
| *Predictors* | *β* | *CI* | *p* | *β* | *CI* | *p* | *β* | *CI* | *p* | *β* | *CI* | *p* | *β* | *CI* | *p* |
| (Intercept) | -0.7 | -0.94 – -0.46 | **<0.001** | -0.71 | -0.95 – -0.47 | **<0.001** | 0.09 | -0.28 – 0.46 | 0.626 | -0.4 | -0.64 – -0.16 | **0.001** | -0.34 | -0.58 – -0.11 | **0.005** |
| APOE [E4] | 0.14 | 0.03 – 0.26 | **0.013** | 0.07 | -0.04 – 0.18 | 0.221 | 0.13 | -0.03 – 0.29 | 0.112 | 0.07 | -0.04 – 0.18 | 0.205 | 0.05 | -0.07 – 0.16 | 0.422 |
| BMI | 0.18 | 0.14 – 0.23 | **<0.001** | 0.2 | 0.15 – 0.25 | **<0.001** | 0.21 | 0.14 – 0.28 | **<0.001** | -0.11 | -0.16 – -0.06 | **<0.001** | 0.25 | 0.21 – 0.30 | **<0.001** |
| Sex [women] | 0.15 | 0.06 – 0.24 | **0.001** | 0.2 | 0.11 – 0.28 | **<0.001** | 0.09 | -0.03 – 0.21 | 0.158 | -0.07 | -0.15 – 0.02 | 0.117 | 0.1 | 0.01 – 0.19 | **0.023** |
| Age [in yrs] | 0.01 | 0.01 – 0.01 | **<0.001** | 0.01 | 0.01 – 0.02 | **<0.001** | 0 | -0.01 – 0.01 | 0.869 | 0.01 | 0.00 – 0.01 | **0.002** | 0 | 0.00 – 0.01 | **0.043** |
| Currently ill | 0.02 | -0.13 – 0.17 | 0.773 | -0.03 | -0.18 – 0.13 | 0.744 | 0.15 | -0.09 – 0.39 | 0.229 | -0.09 | -0.25 – 0.07 | 0.263 | 0.07 | -0.07 – 0.20 | 0.359 |
| Season [wet] | -0.12 | -0.20 – -0.04 | **0.004** | -0.29 | -0.38 – -0.21 | **<0.001** | -0.28 | -0.44 – -0.13 | **<0.001** | 0.06 | -0.03 – 0.15 | 0.168 | 0.11 | 0.04 – 0.18 | **0.003** |
| E4 * BMI | -0.08 | -0.18 – 0.02 | 0.111 | -0.09 | -0.19 – 0.00 | 0.061 | 0.02 | -0.13 – 0.17 | 0.786 | -0.08 | -0.18 – 0.01 | 0.093 | 0.01 | -0.09 – 0.11 | 0.865 |
| **Random Effects** | | | | | | | | | | | | | | | |
| Residual σ^2^ | 0.7 | | | 0.71 | | | 0.3 | | | 0.82 | | | 0.58 | | |
| τ_00_ | 0.22 _pid_ | | | 0.17 _pid_ | | | 0.61 _pid_ | | | 0.13 _pid_ | | | 0.30 _pid_ | | |
|  | 0.08 _community_id_ | | | 0.10 _community_id_ | | | 0.10 _community_id_ | | | 0.10 _community_id_ | | | 0.05 _community_id_ | | |
| ICC | 0.3 | | | 0.27 | | | 0.7 | | | 0.22 | | | 0.38 | | |
| N | 1168 _pid_ | | | 1154 _pid_ | | | 907 _pid_ | | | 1166 _pid_ | | | 1173 _pid_ | | |
|  | 71 _community_id_ | | | 71 _community_id_ | | | 68 _community_id_ | | | 71 _community_id_ | | | 71 _community_id_ | | |
| Observations | 2581 | | | 2390 | | | 1032 | | | 2476 | | | 2632 | | |
| Marginal R^2^ / Cond. R^2^ | 0.049 / 0.336 | | | 0.074 / 0.326 | | | 0.070 / 0.724 | | | 0.027 / 0.239 | | | 0.072 / 0.428 | | |

**Supplementary File 1i.** Full models evaluating the moderating effects of APOE genotype on associations between BMI and cholesterols. Results report both fixed and random effects estimates from mixed effects linear regressions, which include random effects for ID and community residence. In addition to age, sex, and season, a dummy variable was used as a proxy for current illness (leukocytes > 12 mm^3^). Results are reported as standardized betas; *CI* is the 95% confidence interval. All dependent variables were transformed and centered prior to analyses. See methods section for transformations. *APOE* genotype is coded as a categorical variable, binned as individuals that are homozygous *E3* (E3) versus those that have at least one copy of the *E4* allele (E4).
