# Supplementary material for: APOE4 is associated with elevated blood lipids and lower levels of innate immune biomarkers in a tropical Amerindian subsistence population
Source: eLife. 2021 Sep 29;10:e68231. doi: 10.7554/eLife.68231 (PMC8480980; doi:10.7554/eLife.68231)
Supplement: Supplementary file 2. — Link to the Spanish version of the article: https://tsimane.anth.ucsb.edu/results.html#pubs. [file elife-68231-supp2.docx]

Resumen

En contextos post-industriales, el gen de APOE4 está asociado con mayor riesgo de enfermedad cardiovascular y neurológica. Sin embargo, la mayoría de la historia evolutiva sucedió en contextos de alta diversidad patogénica y bajo riesgo cardiovascular. Nosotros planteamos que en contextos de energía limitada y alta carga patógena, los alelos del gen APOE4 son beneficiosos al reducir la inflamación innata cuando no están cursando una infección, al mantener los niveles de lípidos altos que minimizan los altos costos de la activación inmunitaria durante la infección. Entre los agricultores recolectores Tsimane de Bolivia (N=1266, 50% mujeres), el gen APOE4 está asociado con niveles 30% más bajos que la proteína C reactiva, y niveles más altos de colesterol total y LDL oxidados. Los lípidos en sangre fueron no asociados o fueron negativamente asociados con los biomarcadores inflamatorios, excepto por las asociaciones de la LDL oxidado y de la inflamación que estuvieron limitados a los obesos adultos. Además, los portadores del gen APOE4 mantienen niveles altos de colesterol total y LDL en personas con bajo índice de masa corporal. Estos resultados sugieren que las relaciones entre el gen APOE4 y los lípidos pueden ser beneficiosos para la respuesta inmune dependiente de los patógenos y es improbable que aumenten el riesgo cardiovascular en una población altamente activa y de subsistencia. Link a la versión en español del artículo: https://tsimane.anth.ucsb.edu/results.html#pubs
